# LiF‐Dominated SEI Formation via a Lychee‐Like Primary Interphase for Fast‐Charging Natural Graphite Anodes

**DOI:** 10.1002/smll.202504255

**Published:** 2025-07-07

**Authors:** Xiangqi Liu, Qitao Shi, Jiaqi Wang, Junjin Zhang, Cheng Zhang, Zhipeng Wang, Luwen Li, Alicja Bachmatiuk, Yanbin Shen, Ruizhi Yang, Mark H. Rümmeli

**Affiliations:** ^1^ Soochow Institute for Energy and Materials Innovation, College of Energy, Key Laboratory of Advanced Carbon Materials and Wearable Energy Technologies of Jiangsu Province, Key Laboratory of Core Technology of High Specific Energy Battery and Key Materials for Petroleum and Chemical Industry Soochow University Suzhou 215006 China; ^2^ i‐Lab, CAS Center for Excellence in Nanoscience Suzhou Institute of Nano‐Tech and Nano‐Bionics (SINANO) Chinese Academy of Sciences (CAS) Suzhou 215123 China; ^3^ Faculty of Chemistry Wroclaw University of Science and Technology Wybrzeze Wyspiarskiego 27 Wroclaw 50–370 Poland; ^4^ Electron Beam Emergent Additive Manufacturing (EBEAM Centre), Centre for Nanotechnology (CNT), Centre for Energy and Environmental Technologies (CEET) VSB—Technical University of Ostrava 17 Listopadu 15 Ostrava 708 33 Czech Republic; ^5^ Institute for Materials Chemistry IFW Dresden 20 Helmholtz Strasse 01069 Dresden Germany

**Keywords:** fast‐charging anode, LiF‐dominated SEI, lychee‐like primary interphase, natural graphite

## Abstract

Natural graphite, with its lower production cost, higher capacity, and superior electrical conductivity than artificial graphite, currently accounts for approximately 40% of the global lithium‐ion battery anode market. However, the inadequate compatibility of natural graphite with commercial carbonate ester electrolytes leads to irreversible capacity loss, reduce coulombic efficiency, and rapid capacity decline during cycling. Applying an oxygen‐deficient titanium dioxide (TiO_2‐x_) protective layer to natural graphite anodes has been noted as a successful method for improving their structural integrity and cycling stability; however, the fragile solid–electrolyte interphase (SEI) limits their fast‐charging capability. In this study, nitrogen atoms are strategically incorporated into the TiO_2‐x_ surface structures, creating a lychee‐like primary interphase that regulated the interfacial electrochemistry and facilitated the development of a LiF‐dominated SEI. The robust LiF‐dominated SEI, as examined through ex situ X‐ray photoelectron spectroscopy analysis and kinetic evaluations, successfully mitigates interfacial side reactions and enhances bulk charge transfer. Consequently, the modified natural graphite anodes exhibit improved capacities at higher current densities, delivering a stable reversible capacity of 388.9 mAh g^−1^ after 200 cycles at a rate of 5 C.

## Graphical abstract

This study enhances fast‐charging natural graphite anodes by creating a lychee‐like primary interphase. This structure promotes the formation of a robust, LiF‐dominated solid electrolyte interphase (SEI). The modified anode exhibits significantly improved kinetics and stability, delivering a high reversible capacity of 388.9 mAh/g after 200 cycles at 5 C by suppressing side reactions and facilitating Li^+^ transport.

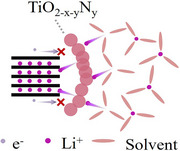

## 1 Introduction

The goal of achieving “carbon neutrality and peaking emissions” has driven rapid advancement in new energy vehicles and cutting‐edge energy storage technologies, significantly boosting the market share of Lithium‐Ion batteries (LIBs).^[^ ^1^ ^]^ In conventional LIB configurations, the anode plays an essential role as a key repository for the intercalation and deintercalation of lithium (Li) ions, directly influencing the energy capacity, longevity, and rate performance of LIBs.^[^ ^2^, ^3^ ^]^ Since the successful commercialization of LIBs in the 1990s, graphite anodes have remained the predominant choice among various anode materials.^[^ ^4^, ^5^ ^]^ Natural Graphite (NG) has emerged as an ideal anode material for LIBs owing to its abundant reserves, low production costs, high capacity, and excellent electrical conductivity. However, the limited van der Waals forces in graphite layers and the anisotropic transport of Li ions within these graphene layers present several challenges.^[^ ^6^ ^]^

In carbonate‐based electrolytes, the strong bond between Li ions and solvent molecules result in slow desolvation, leading to solvent co‐intercalation, which causes expansion, cracking, and exfoliation of the graphitic layers. Moreover, side reactions related to parasitic reduction at the electrode/electrolyte interface result in a dense Solid Electrolyte Interphase (SEI),^[^ ^7^, ^8^ ^]^ affecting the safety, low‐temperature performance, and fast charging capability of LIBs. Interface modification has become a popular method for enhancing the fast charging capability and durability of graphite anodes. Incorporating amorphous carbon,^[^ ^9^, ^10^ ^]^ metal oxides,^[^ ^11^ ^]^ and other materials to create a core–shell configuration offers several advantages, such as a reduced specific surface area of graphite and a decrease in electrolyte usage. Additionally, core–shell configuration in LIBs safeguards against direct interactions between the graphite layers and electrolytes, thereby improving battery stability. Furthermore, the shell structure reduces the orientation selectivity of Li^+^ intercalation in NG anodes.

Titanium Dioxide (TiO_2_), known for its low thermal expansion, high mechanical stability, and excellent electrochemical reversibility, holds great promise as a composite coating material. However, the relatively large band gap (3.2 eV) of TiO_2_ results in low conductivity.^[^ ^12^, ^13^ ^]^ Previous studies have explored various strategies, including reducing the material size, designing nanostructures,^[^ ^14^, ^15^, ^16^, ^17^ ^]^ introducing advanced conductive agents,^[^ ^18^, ^19^ ^]^ doping with heteroatoms,^[^ ^20^, ^21^, ^22^, ^23^, ^24^, ^25^ ^]^ and creating oxygen vacancies or incorporating trivalent titanium^[^ ^26^, ^27^, ^28^ ^]^ to enhance material performance. Metal oxides, such as oxygen‐deficient TiO_2_^[^ ^29^ ^]^ are often applied to NG surfaces to address the swift voltage drop in graphite anodes during fast charging in LIBs. Note that TiO_2‐x_ coatings can reduce the electrode–electrolyte interfacial resistance, potentially enhancing the fast‐charging capabilities of graphite anodes. However, the presence of bulk TiO_2_ limits the fast charging performance of LIBs.

This study investigated nitrogen‐doped TiO_2‐x_, structured like alychee, as a primary interphase on NG surfaces to improve LIB performance by mitigating the structural damage to graphite. The observed improvement in Li‐ion transport kinetics ensured the stable operation of graphite anodes. A uniform TiO_2_ coating was first applied to an NG surface, followed by nitrogen doping of TiO_2_ (TiO_2‐x‐y_N_y_) via ammonium chloride treatment at high temperatures, resulting in a surface layer resembling the outer skin of a lychee. Note that nitrogen doping introduces impurity states into the TiO_2‐x_ bandgap. The presence of mid‐gap impurity states narrows the bandgap, forms valence and conduction bands, increases the electronic conductivity, and promotes Li‐ion transport rates.^[^ ^30^ ^]^ The lychee‐like primary interphase reduced the interfacial resistance between the electrode and electrolyte, improved the Li‐ion transport dynamics, and ultimately enhanced the cyclic stability and rate performance of the examined TiO_2_‐based electrode. The fabricated TiO_2‐x‐y_N_y_@NG anode demonstrated excellent performance, achieving a reversible capacity of 388.9 mAh g^−1^ over 200 cycles at a rate of 5 C. Ex situ bulk and interfacial analysis revealed that bare NG experiences severe structural damage, whereas the interface modification strategy proposed in this study effectively suppresses the damage to NG by forming a LiF‐dominated SEI.

## 2 Results and Discussion

### 2.1 Morphological Analysis

**Figure 1:**
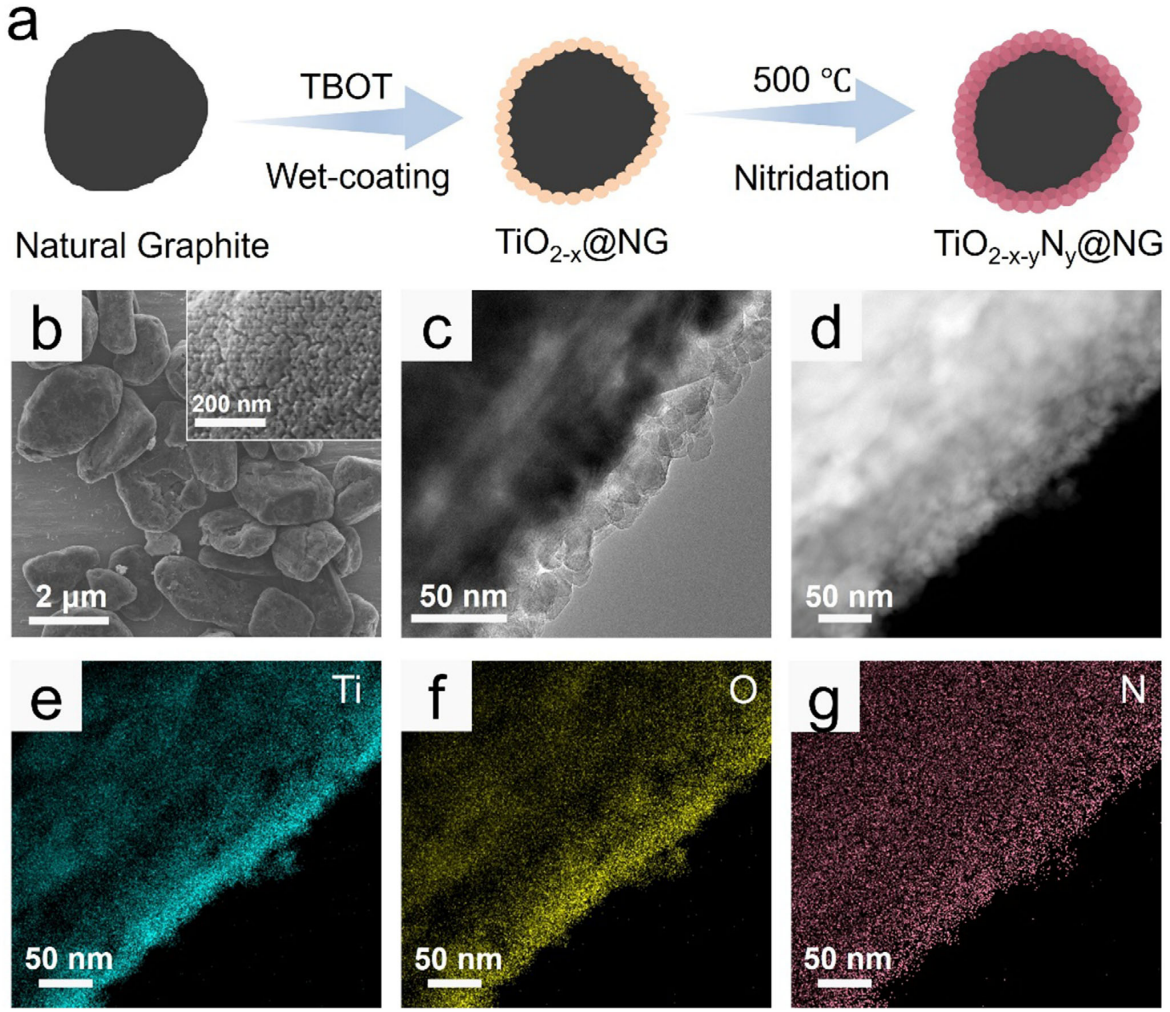
a) Schematic of the synthesis procedure for TiO_2‐x‐_N_xy_N_y_@NG. b) SEM and c) TEM images of TiO_2‐x‐_N_xy_N_y_@NG. d) STEM image (captured in HAADF mode) images of Figure 1. (a) Schematic of the procedure used for synthesizing TiO_2‐x‐y_N_y_@NG. (b) SEM (c) TEM and (d) STEM TiO_2‐x‐y_N_y_@NG. EDS maps showing distributions of e) Ti, f) O, and g) N within the area shown in (d).

**Figure** **1a** illustrates the synthesis of TiO_2‐x‐y_N_y_@NG via Tetrabutyl titanate (TBOT) hydrolysis to form a TiO_2‐x_ coating, followed by NH_4_Cl treatment to induce nitrogen doping in the TiO_2‐x_ interphase. The wet synthesis method and subsequent NH_4_Cl vapor treatment at moderate temperatures facilitated large‐scale production at a low cost. The bulk morphologies of NG, TiO_2‐x_@NG, and TiO_2‐x‐y_N_y_@NG are similar, as shown in Figures 1b and S1 (Supporting Information). Scanning electron microscopy (SEM) images in Figure S1 (Supporting Information) show that the NG particles exhibit a smooth surface. After TiO_2‐x_ coating, the particles present a rough surface layer. Following the NH_4_Cl vapor treatment, the surface coating layer becomes even rougher, exhibiting a morphology resembling that of a lychee shell. Further examination of the particle coating layer using transmission electron microscopy (TEM) revealed that the TiO_2‐x_@NG particles possess a uniform TiO_2‐x_ coating layer with a thickness of approximately 15 nm (Figure S2a, Supporting Information). After high‐temperature nitridation, the obtained TiO_2‐x‐y_N_y_ crystals were larger than the TiO_2‐x_ crystals, and the coating layer became porous and thickened to approximately 25 nm (insets in Figure 1b,c). Next, energy‐dispersive X‐ray (EDX) spectrometry was employed to investigate the elemental distribution in the coated layer. The EDX maps (Figure 1d–g) reveal similar elemental distributions, with distributions of Ti, O, and N atoms closely matching the shape of the NG core, indicating successful nitrogen doping and homogeneous coverage of the NG by the TiO_2‐x‐y_N_y_ coating layer.

### 2.2 Chemical Analysis

**Figure 2:**
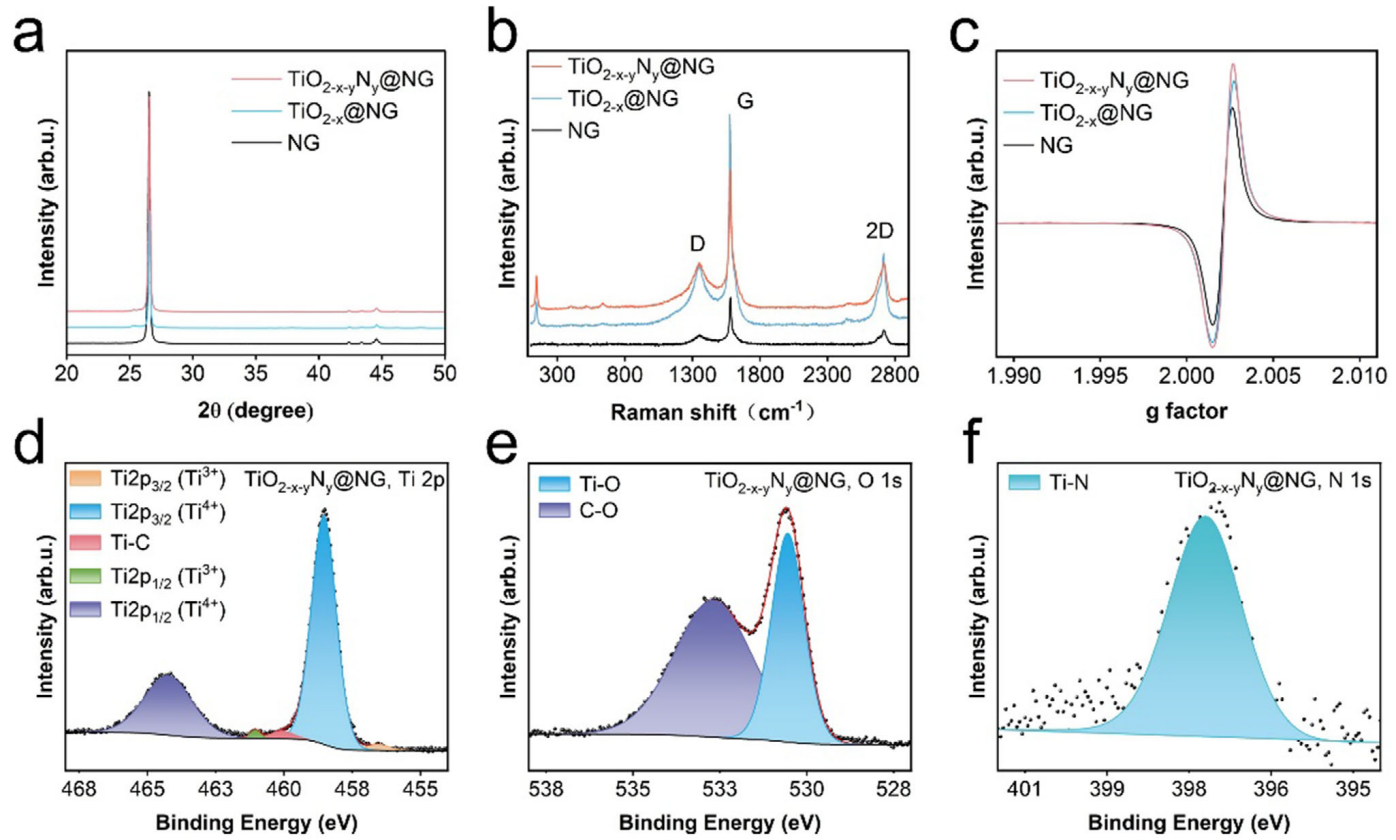
a) XRD patterns, b) Raman spectra, and c) EPR spectra of NG, TiO_2‐x_@NG, and TiO_2‐x‐y_N_y_@NG. XPS spectra of d) Ti 2p, e) O 1s, and f) N 1s core levels in TiO_2‐x‐y_N_y_@NG.

The structural characteristics of pristine and modified NGs were assessed using X‐ray diffraction (XRD). As shown in **Figure** **2**a, all samples exhibit almost indistinguishable XRD peaks. The peaks observed at approximately 26.4°, 42.2°, 43.4°, and 44.4°, aligning with (002), (100), (101), and (102) graphitic planes, respectively,^[^ ^31^ ^]^ indicate that the modified NG retained its original crystalline structure.^[^ ^32^ ^]^ The XRD patterns of both TiO_2‐x_@NG and TiO_2‐x‐y_N_y_@NG show peaks at approximately 25.3°,^[^ ^29^ ^]^ which can be attributed to TiO_2‐x_. Notably, the intensity of the TiO_2‐x‐y_N_y_@NG‐related peak is slightly higher than that of the TiO_2‐x‐y_N_y_@NG‐related peak (Figure S3, Supporting Information). Next, Raman spectroscopy was performed to gain additional information regarding the structural stability of the examined NG samples. As depicted in Figure 2b, both pristine and modified NGs exhibit characteristic D (linked to defect‐related modes in graphene) and G bands (associated with E_2g2_ vibrational mode of graphite) Raman bands at approximately 1350 cm⁻¹ and 1580 cm⁻¹, respectively.^[^ ^33^, ^34^, ^35^, ^36^ ^]^ The D band is associated with irregularities in the arrangement of carbon atoms within the graphite lattice, whereas the G band is associated with the oscillation of sp^2^‐bonded carbon atoms.^[^ ^37^ ^]^ The D band to G band intensity ratio (I_D_/I_G_) serves as a crucial measure of the structural irregularities in carbon materials, with elevated I_D_/I_G_ values indicating increased surface imperfections.^[^ ^38^ ^]^ As shown in Figure 2b, the I_D_/I_G_ ratios for NG, TiO_2‐x_@NG, and TiO_2‐x‐y_N_y_@NG are 0.18, 0.29, and 0.33, respectively, suggesting that lattice defects increase after the high‐temperature treatment and nitrogen doping process. The additional Raman peaks observed at 144 cm^−1^, 406 cm^−1^, 508 cm^−1^, and 631 cm^−1^ in the Raman spectra of TiO_2‐x_@NG and TiO_2‐x‐y_N_y_@NG are absent in the Raman spectrum of pristine NG (Figure S4, Supporting Information). The additional peaks in the Raman spectra of TiO_2‐x_@NG and TiO_2‐x‐y_N_y_@NG originate from the TiO_2_.^[^ ^39^ ^]^ Next, electron paramagnetic resonance (EPR) spectra were recorded to gain further insights into the defect states. As shown in Figure 2c, NG, TiO_2‐x_@NG, and TiO_2‐x‐y_N_y_@NG exhibit defect peaks at g = 2.002. The defect concentration follows the order NG < TiO_2‐x_@NG < TiO_2‐x‐y_N_y_@NG, The relatively higher EPR signal intensity from TiO_2‐x_@NG can be attributed to oxygen vacancies, and the additional increase in EPR signal intensity from TiO_2‐x‐y_N_y_@NG can be attributed to nitrogen doping‐induced oxygen vacancies. The EPR results are consistent with the Raman spectroscopy results. Next, X‐ray photoelectron spectroscopy (XPS) was employed to analyze the chemical composition of the TiO_2‐x‐y_N_y_@NG coating and confirm the successful incorporation of nitrogen. As shown in Figure 2d, the Ti 2p spectrum of TiO_2‐x‐y_N_y_@NG exhibits Ti^3+^‐related peaks at 461.05 eV and 456.57 eV,^[^ ^40^ ^]^ confirming the presence of trivalent titanium. The N 1s spectrum (Figure 2f) shows a peak at 397.92 eV, which corresponds to nitrogenated Ti, providing further evidence of successful nitrogen doping in TiO_2_. The observation of XPS signals related to Ti^3+^ suggests improved electronic conductivity of TiO_2‐x‐y_N_y_@NG compared to that of TiO_2‐x_@NG.^[^ ^41^ ^]^ Note that the N 1s XPS spectrum of TiO_2‐x_@NG shows no detectable N‐related signal (Figure S5, Supporting Information). A comparison of the O1s spectra of TiO_2‐x_@NG and TiO_2‐x‐y_N_y_@NG shows a lower proportion of Ti–O bonds in TiO_2‐x‐y_N_y_@NG (Figure 2e). The reduction in Ti–O bonds can be attributed to the substitution of oxygen atoms with nitrogen during doping.

### 2.3 Electrochemical Analysis

**Figure 3:**
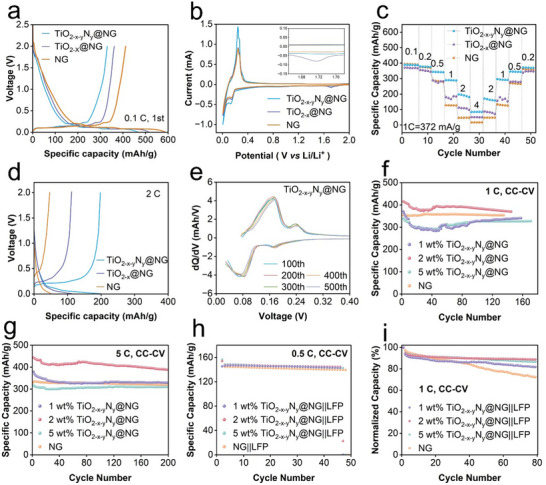
a) Initial charge/discharge curves at 0.1 C. b) Initial CV curves at 0.1 mV s^−1^. c) Rate performance. d) Comparison of charge/discharge curves at 2 C. e) dQ/dV curve of the TiO_2‐x‐y_N_y_@NG anodes. Cycling performance at f) 1 C and g) 5 C in CC‐CV mode the TiO_2‐x‐y_N_y_@NG anodes. Cycling performance of full batteries in a voltage range of 2.4‐3.8 V at charge/discharge rate of h) 0.5 C/0.5 C, and i) 0.5 C/1 C.

At 0.1 C, the NG electrode exhibited initial discharge and charge capacities of 542.7 mAh g^−1^ and 381.7 mAh g^−1^, respectively. Similarly, the TiO_2‐x_@NG electrode exhibited initial discharge and charge capacities of 521.7 mAh g^−1^ and 361.4 mAh g^−1^, respectively. In contrast, the TiO_2‐x‐y_N_y_@NG electrode exhibited relatively low discharge and charge capacities of 480.0 mAh g^−1^ and 330.5 mAh g^−1^, respectively (**Figure** **3**a). The initial coulombic efficiencies (ICEs) of the NG, TiO_2‐x_@NG, and TiO_2‐x‐y_N_y_@NG anodes were determined as 70.3%, 69.3%, and 68.8%, respectively. The observed sequential decrease in ICE among the examined materials can be attributed to highly reactive carbon defects that interact with Li^+^ ions and form inactive Li, which, in turn, reduces the ICE. Additionally, the active components within the primary interphase may react with Li^+^ ions in the electrolyte, deplete some Li^+^ ions and subsequently reduce the number of active Li^+^ ions, ultimately lowering the ICE. The observed decrease in ICE can also be associated with an increased specific surface area. Brunauer–Emmett–Teller (BET) analysis revealed that NG exhibits a type III adsorption/desorption isotherm, whereas TiO_2‐x_@NG and TiO_2‐x‐y_N_y_@NG exhibit type IV adsorption/desorption isotherms (Figure S6, Supporting Information), indicating the formation of mesopores after coating. The presence of mesopores often enhances Li‐ion transport within battery electrode materials. The high porosity and specific surface area provided abundant diffusion pathways and active sites, improving the reaction kinetics. A low ICE of the coating material adversely affects the overall electrode performance. Additionally, the low reversible capacity of the coating material well explains the observed decline in the first cycle assessing the specific capacity of the modified electrode (Figure S7, Supporting Information). Notably, the modified NG retains the Li insertion behavior of NG (Figure S8, Supporting Information). Next, cyclic voltammetry (CV) measurements were performed to gain insights into the electrochemical behavior of the examined electrodes. The initial discharge peaks at approximately 1.7 V indicate the formation of an SEI film at the graphite–electrolyte boundary. The observed shoulder peaks possibly arise from a multiphase lithiation/delithiation process within graphite. The CV curves in Figure 3b show that all oxidation peaks exhibit sharp contours, indicating that the coating layers do not hinder Li^+^ insertion/extraction within the graphite layer structure. The observation that the TiO_2‐x‐y_N_y_@NG anode exhibits a higher peak current than those exhibited by the TiO_2‐x_@NG and NG anodes indicated that lychee‐like primary interphase promotes faster Li^+^ diffusion and increases reversible capacity.

Next, the fabricated electrodes were tested at a high current density of 1 C to evaluate their fast‐charging capability. The NG electrode exhibited an initial capacity of 257.6 mAh g^−1^ at 1 C and a capacity of 167.2 mAh g^−1^ after 500 cycles. In contrast, the TiO_2‐x‐y_N_y_@NG electrode exhibited a high reversible capacity of 249.3 mAh g^−1^ even after 500 cycles, with a capacity retention of 109.3% (Figure S9, Supporting Information). As the discharge/charge rate was increased stepwise from 0.1 C to 4 C, the TiO_2‐x‐y_N_y_@NG anode consistently exhibited the highest discharge capacity among all the examined electrodes at all stages (Figure 3c). Notably, at a rate of 2 C, the TiO_2‐x‐y_N_y_@NG anode exhibited an excellent capacity of 199.0 mAh g^−1^, significantly surpassing the capacities exhibited by the NG (47.1 mAh g^−1^) and TiO_2‐x_@NG (113.3 mAh g^−1^) anodes (Figure 3d), demonstrating excellent Li^+^ transportation kinetics.

At a rate of 0.2 C, the specific capacities exhibited by the modified NG anodes were relatively lower during the first 40 cycles (Figure S10, Supporting Information), possibly because the Li storage within the coating material, either TiO_2‐x_ or TiO_2‐x‐y_N_y_, affects the overall capacity. As the number of cycles increased, the NG anode exhibited a rapid capacity decay owing to SEI thickening and structural degradation. In contrast, the TiO_2‐x‐y_N_y_@NG anode exhibited a significantly lower rate of capacity decay over long‐term cycling. Incremental capacity analyses (dQ/dV) were performed to assess electrochemical reaction kinetics and electrode stability, and the results are presented in Figures 3e and S10 (Supporting Information). The peaks observed during the cycling of the NG electrode shifted in position and gradually decreased in intensity, indicating a loss of electrochemical activity and a reduction in the stability of graphite. In contrast, the TiO_2‐x‐y_N_y_@NG anode exhibited well‐aligned dQ/dV curves without significant peak shifts or intensity reductions, highlighting the enhanced stability of both the SEI and bulk structure.

To determine the optimal coating amount, a series of modified NG anodes with varying concentrations of TiO_2‐x‐y_N_y_ (Figure S12–S14, Supporting Information) were prepared and examined via comprehensive electrochemical tests. The anode with 2 wt.% coating demonstrated the highest specific capacity and superior cycling performance. In the constant current‐constant voltage (CC‐CV) tests performed at 1 C, the modified electrode with 2 wt.% coating exhibited a remarkable reversible capacity of 373.1 mAh g^−1^ after 135 cycles, significantly outperforming the pristine NG anode, which only exhibited a capacity of 353.8 mAh g^−1^ (Figure 3f). Notably, the modified electrode exhibited a “negative capacity decay” phenomenon, which is consistent with previous experimental observations,^[^ ^42^ ^]^ indicating continuous capacity enhancement during cycling.

To evaluate the stable cycling performance under extreme charging conditions, comparative studies were conducted between pristine graphite electrodes and TiO_2‐x‐y_N_y_@NG composite electrodes with TiO_2‐x‐y_N_y_ contents ranging from 1 to 5 wt.% under a high current density of 1860 mA g^−1^ (Figure 3g). The anode with 2 wt.% coating exhibited the highest capacity retention, exhibiting a capacity of 388.9 mAh g^−1^ even after 200 cycles, whereas the pristine NG anode exhibited a capacity of 319.7 mAh g^−1^. Conversely, the anode with 5 wt.% coating exhibited a decrease in specific capacity owing to the inherently low capacity of the coating material. Notably, the electrochemical characteristics of the anode with 1 wt.% coating were similar to those exhibited by the pristine NG anode, likely owing to the insufficient coverage of the active material surface. Generally, technologies capable of achieving full charge or 80% within 30 minutes (charge/discharge rates ≥ 2 C) are recognized as fast‐charging systems.^[^ ^43^ ^]^ When discharged for 30 minutes, the anode with 2 wt.% coating exhibited a capacity of 363.2 mAh g^−1^, whereas the anode with 5 wt.%, 1 wt.% and pristine NG anode respectively exhibited a capacity of 304.8, 316.5, 293.9 mAh g^−1^. The obtained results highlight that the amount of coating applied to an NG anode material significantly impacts its electrochemical performance in fast‐charging batteries. Particularly, a lychee‐like primary interphase structure significantly enhances the fast charging capability of anode materials in LIBs.

To evaluate the practicality of the TiO_2‐x‐y_N_y_@NG anode, full cells are assembled by pairing with LFP cathodes. Under a charge/discharge rate of 0.5 C/0.5 C, the 2 wt.% TiO_2‐x‐y_N_y_@NG||LFP full cell demonstrated a discharge capacity of 146.6 mAh g^−1^ at the 44th cycle. In comparison, the cells with 5 wt.%, 1 wt.%‐coated and pristine NG anodes exhibited lower capacities of 143.0 mAh g^−1^, 145.2 mAh g^−1^, and 140.1 mAh g^−1^, respectively, under the same testing conditions (Figure 3h). Notably, a charge/discharge rate of 0.5 C/1 C charge rate, the 2 wt.% TiO_2‐x‐y_N_y_@NG||LFP configuration maintained superior cycling stability, retaining 89.1% of its initial capacity after 80 cycles. In contrast, the NG||LFP full cell showed significant capacity degradation and inferior cyclability under identical conditions (Figure 3i).

### 2.4 Kinetics Analysis

**Figure 4:**
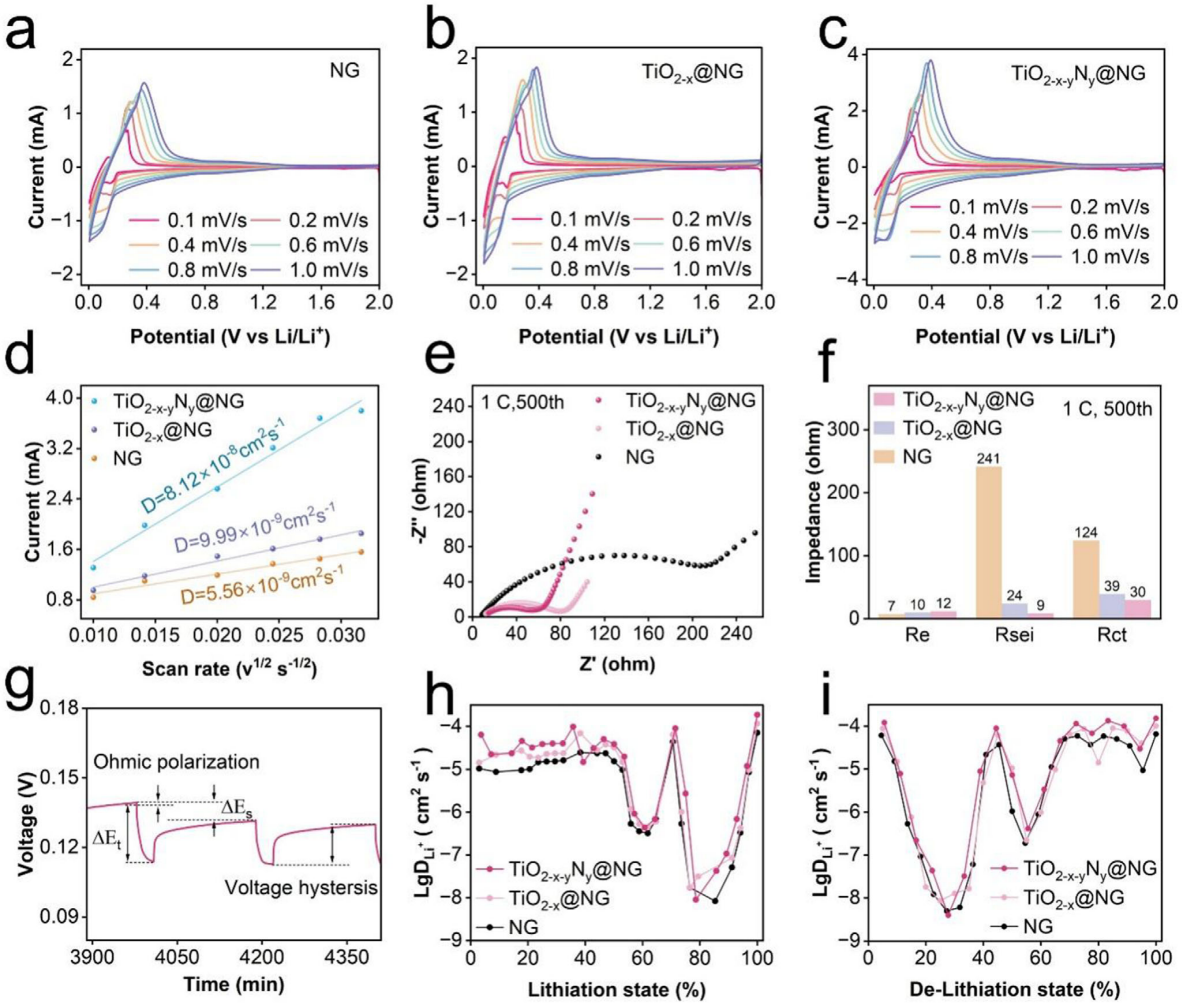
CV curves of a) NG, b) TiO_2‐x_@NG, and c) TiO_2‐x‐y_N_y_@NG anodes at different scan rates. d) Li‐ion diffusion coefficient of NG, TiO_2‐x_@NG, and TiO_2‐x‐y_N_y_@NG anodes. e) Nyquist plots of various anodes. f) Associated resistances: electrolyte resistance (R_e_), SEI resistance (R_SEI_), and charge transfer resistance (R_ct_). g) Detailed GITT voltage–time profile. Li‐ion diffusion coefficient during h) lithiation and i) de‐lithiation processes.

The CV curves of the electrodes obtained at different scan rates ranging from 0.1 to 1 mV s^−1^ (**Figure** **4**a–c) show that the integrated area under the oxidation and reduction peaks increase with increasing scan rate. As the scan rate increases, the separation between the oxidation and reduction peaks become more pronounced, possibly because of increased electrode polarization under higher current conditions. The diffusion coefficient of Li^+^ ions can be determined using the following equation:^[^ ^44^ ^]^

(1) $$\;I_{p}=2.69\times 10^{5}n^{3/2}AD^{1/2}v^{1/2}C\;$$

The peak current, denoted as Ip, is influenced by several key factors: (i) scan rate (v) measured in volts per second, (ii) total number of electrons (n) participating in the electrochemical reaction, (iii) electrode surface area (A) in square centimeter, (iv) diffusion coefficient (D) within the electrode, and (v) surface concentration (C) of the electrode material. The Li⁺ diffusion coefficient exhibited by the TiO_2‐x‐y_N_y_@NG anodes (8.12 × 10^−8^ cm^2^ s^−1^) is significantly higher than that exhibited by the NG anodes (5.56 × 10^−9^ cm^2^ s^−1^), as shown in Figure 4d.

(R_SEI_), charge transfer resistance (R_ct_), and solid‐state The Nyquist plots were constructed to show the impedance values of the NG, TiO_2‐x_@NG, and TiO_2‐x‐y_N_y_@NG anodes. As shown in Figure 4e and f, the Nyquist plots show an x‐axis intersection, a semicircular arc, and a descending linear trend across the different frequency ranges, corresponding to the electrolyte resistance (R_e_), SEI resistance Li^+^ diffusion process.^[^ ^45^ ^]^ The Nyquist plots reveal that the R_SEI_ and R_ct_ values for the TiO_2‐x‐y_N_y_@NG anode are significantly lower than those of pristine NG anode indicating a more efficient activation process for the TiO_2‐x‐y_N_y_@NG anode.

Next, the diffusion of Li^+^ ions within the solid electrode, which is the rate‐determining step in the electrochemical process, was analyzed using the galvanostatic intermittent titration technique (GITT; Figure S15, Supporting Information). Assuming that Li^+^ diffusion occurs according to Fick's second law, the diffusion coefficient (D_Li_) is determined using the following equation:^[^ ^46^ ^]^

(2) $$\;D_{\mathrm{Li}}=\frac{4}{\pi \tau }\;{\left(\frac{m_{B}V_{M}}{M_{B}S}\right)}^{2}{\left(\frac{\Delta E_{S}}{\Delta E_{\tau }}\right)}^{2}\;$$

where τ denotes the fixed duration of the constant current discharge. m_B_, V_M_, and M_B_ represent the mass, molar volume, and molar mass of the active material, respectively. ΔE_S_ and ΔE_τ_ represent the shifts in steady‐state voltage and overall battery voltage, respectively; these shifts are observed throughout the discharge process, as illustrated in Figure 4g. As illustrated in Figure 4h and i, the TiO_2‐x‐y_N_y_@NG anode exhibits a higher Li^+^ diffusion coefficient than those exhibited by the NG and TiO_2‐x_@NG anodes at all lithiation and de‐lithiation stages. The enhanced Li⁺ diffusion kinetics are attributed to the mesoporous structure shortening Li⁺ transport pathways and reducing solid‐state diffusion barriers, while the introduction of a lychee‐like TiO_2‐x‐y_N_y_ primary interphase improves electronic conductivity and accelerates charge transfer at the electrode‐electrolyte interface.

### 2.5 Ex Situ Characterizations

**Figure 5:**
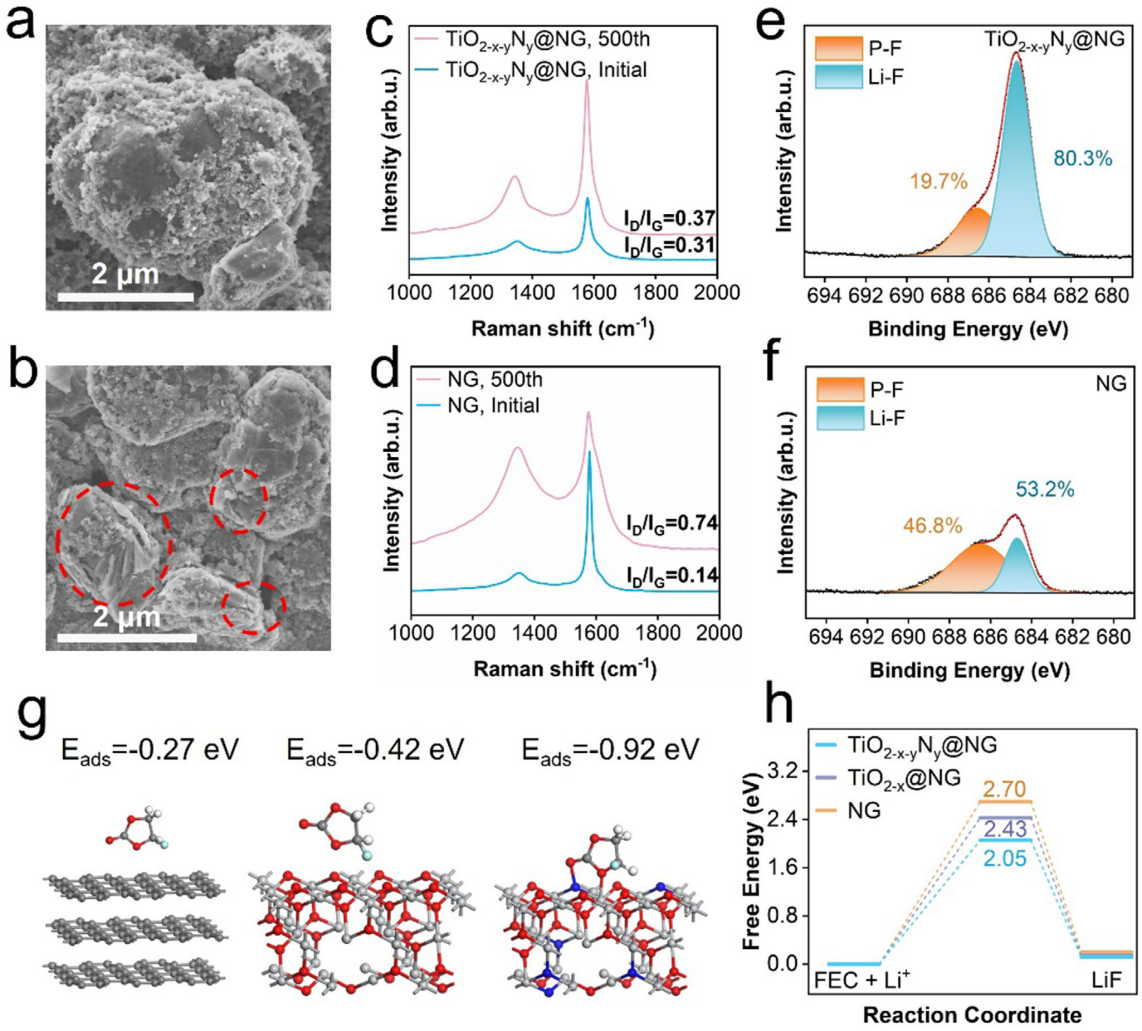
SEM images of a) TiO_2‐x‐y_N_y_@NG and b) NG anodes after 500 cycles at 1 C. Ex situ Raman spectra of c) TiO_2‐x‐y_N_y_@NG and d) NG anodes before and after 500 cycles at 1 C. XPS F 1s spectra of SEI layers on the surfaces of e) TiO_2‐x‐y_N_y_@NG and f) NG anodes after 500 cycles at 1 C. g) Adsorption configurations of FEC molecules on anode surfaces. h) Relative energy distribution of LiF on anode surfaces.

SEM and Raman analyses were conducted on the fabricated electrodes before and after 500 cycles at 1 C to further evaluate the role of the lychee‐like TiO_2‐x‐y_N_y_ primary interphase in enhancing the structural stability of electrodes for LIBs. Internal localized cumulative stress and co‐intercalation of solvated Li^+^ ions can cause severe exfoliation and amorphization of the NG anode during long‐term cycling.^[^ ^47^ ^]^ Cracks and exposed edge planes are observed on the surfaces of the NG and TiO_2‐x_@NG electrodes (**Figures** **5**b and S16, Supporting Information), with an increase in the I_D_/I_G_ ratio after cycling, indicating structural degradation of the electrodes.^[^ ^48^ ^]^ In contrast, the TiO_2‐x‐y_N_y_@NG anode retains its layered structure and exhibits minimal changes in the I_D_/I_G_ ratio, whereas the NG anode exhibits significant structural changes after cycling (Figures 5c,d; S17, Supporting Information). The observed structural degradation after cycling can be attributed to solvent co‐intercalation that leads to poor electrochemical performance. Notably, the TiO_2‐x‐y_N_y_@NG anode maintains a smooth and compact surface even after cycling (Figure 5a), demonstrating that the presence of a lychee‐like TiO_2‐x‐y_N_y_ surface layer provides robust protection and enhances long‐term cycling stability of the TiO_2‐x‐y_N_y_@NG anode. The obtained results confirm that the lychee‐like TiO_2‐x‐y_N_y_ interphase effectively safeguards NG from solvent co‐intercalation and interfacial side reactions, effectively mitigating structural degradation over extended cycling.

Ex situ XPS analysis was conducted to investigate the chemical composition of the SEIs on aged electrodes, providing insights into interfacial electrochemistry. The fluorine content of the SEI layer of the TiO_2‐x‐y_N_y_@NG anode was significantly higher than that of the pristine NG anode (Figure S18, Supporting Information). The F 1s XPS spectrum revealed two primary fluorine‐based components: (i) Li–F (684.6 eV)—derived from the reduction of the fluoroethylene carbonate(FEC) additives; and (ii) Li_x_PO_y_F_z_ (686.5 eV)—an intermediate decomposition product of LiPF_6_.^[^ ^49^ ^]^ Notably, the SEI layer on the aged TiO_2‐x‐y_N_y_@NG anode exhibited a LiF ratio of up to 80.3%, substantially exceeding those exhibited by the cycled NG and TiO_2‐x_@NG anodes (Figure 5e,f; Figure S19, Supporting Information). The LiF‐dominated SEI enhanced interfacial stability by suppressing side reactions and facilitating rapid Li^+^ migration. Additionally, a high shear modulus and interfacial energy of LiF‐dominated SEI^[^ ^50^, ^51^, ^52^ ^]^ effectively protect the graphite structure from internal stresses. The findings of this study demonstrate that TiO_2‐x‐y_N_y_@NG anodes exhibit a robust SEI, structurally stable bulk phase, and superior electrochemical performance.

Theoretical calculations were performed to further validate the experimental results and provide mechanistic insights into LiF‐dominated SEI formation. The density functional theory (DFT) calculations reveal that the lychee‐like TiO_2‐x‐y_N_y_ interphase exhibits the strongest adsorption energy for FEC (−0.92 eV), surpassing those exhibited by the TiO_2‐x_ (−0.42 eV) and pristine NG (−0.27 eV) interphases. The enhanced FEC adsorption exhibited by the TiO_2‐x‐y_N_y_ interphase stems from the synergistic effect of N‐doping and oxygen vacancies, creating abundant Lewis acid sites (Ti^3^⁺‐vacancy pairs) that coordinate with FEC molecules (Figure 5g). Concurrently, the energy barrier for LiF formation on TiO_2‐x‐y_N_y_ interphase is calculated to be 2.05 eV, which is substantially lower than those calculated for TiO_2‐x_ (2.43 eV) and pristine NG (2.70 eV) interphases. A reduction in the energy barrier for LiF formation can be attributed to N‐doping‐induced electron delocalization, which strengthens the interaction between Ti^3^⁺ and F⁻ ions, facilitating LiF nucleation. The results of DFT calculations align well with the XPS results, confirming that the presence of lychee‐like interphase promotes selective FEC decomposition and LiF formation (Figure 5h). The relatively lower values of FEC adsorption energy and LiF formation barrier exhibited by the TiO_2‐x‐y_N_y_@NG interphase create a thermodynamic preference for LiF‐dominated SEI growth, suppressing electrolyte co‐intercalation and structural degradation. The theoretical findings are well supported by experimental findings, confirming a strong link among the lychee‐like interphase architecture, interfacial energetics, and SEI composition.

## 3 Conclusion

A lychee‐like primary TiO_2‐x‐y_N_y_ interphase was successfully synthesized on NG via a facile wet‐chemical strategy, followed by a modest‐temperature nitriding treatment. The TiO_2‐x‐y_N_y_ interphase effectively suppressed undesired side reactions at the electrode–electrolyte interface by facilitating the LiF‐dominated SEI. As a result, the TiO_2‐x‐y_N_y_@NG electrode demonstrated a high reversible capacity of 388.9 mAh g^−1^ after 200 cycles at 5 C. The fast charging capability of the TiO_2‐x‐y_N_y_@NG electrode was comprehensively evaluated using kinetic, bulk, and interfacial characterization methods. Theoretical calculations revealed that the lychee‐like interface exhibits the highest FEC adsorption energy and lowest LiF formation barrier, driving selective SEI composition and suppressing electrolyte co‐intercalation. The lychee‐like primary interphase effectively mitigated the structural degradation of graphite during cycling, reduced interfacial impedance, and enhanced Li‐ion diffusion dynamics. The reported interfacial engineering method presents a viable solution for developing cutting‐edge anode materials for next‐generation high‐energy‐density LIBs.

## 4 Experimental Section

### Synthesis of TiO_2‐x‐y_N_y_@NG Particles

Four grams of tetrabutyl titanate were dispersed in 100 mL of anhydrous ethanol. Subsequently, 5 g of NG was added to the prepared mixture. The mixture was then stirred for 2 h to initiate hydrolytic condensation. After washing with anhydrous ethanol and deionized water, the mixture was dried in an oven. The obtained particles were placed in a tubular furnace and annealed in an argon–hydrogen mixture at 600 °C for 10 h to obtain TiO_2‐x_ coated NG (TiO_2‐x_@NG). The powder containing TiO_2‐x_@NG particles was subsequently placed in a sealed stainless‐steel tube in the presence of an adequate amount of ammonium chloride, and subsequently, the tube was maintained at 500 °C for 2 h to allow the reaction to proceed. After cooling, the obtained products were rinsed with deionized water to eliminate hydrogen chloride. The solid products were collected after drying and labeled as TiO_2‐x‐y_N_y_@NG.

### Structural Characterization

XRD data were recorded using a Bruker D8 Advance diffractometer equipped with a Cu Kα radiation source operating at 40 kV and 40 mA. For ex situ XRD measurements, a cycled button cell was carefully disassembled in a glove box to extract the working electrode. The electrode was thoroughly rinsed with dimethyl carbonate (DMC) to eliminate the residual electrolyte and then air‐dried at ambient temperature. To avoid air exposure, the dried electrode was attached to the XRD sample holder using polyimide tape. SEM images and energy‐dispersive spectroscopy (EDS) maps were recorded using a Hitachi SU8010 SEM operating at 10 kV and 10 mA for SEM and 15 kV and 15 mA for EDS. XPS data were collected using a Thermo Fisher Escalab 250Xi XPS system equipped with an Al Kα radiation source, which was housed in a vacuum transfer chamber to prevent air contamination. In XPS analysis, the binding energies were referenced to the C1s peak at 284.8 eV, which corresponds to carbon contamination from the atmosphere. TEM images were recorded using a spherical aberration‐corrected FEI Titan Themis Cubed G2 300 TEM microscope operating at 300 kV. In addition, the defect densities in the examined materials were assessed using Horiba HR Evolution spectroscopy. The specific surface area and pore size distribution were analyzed using a BET micromeritics instrument (ASAP 2460). Thermal gravimetric analyzer (TGA) (NETZSCH, TG209) was used to identify the residual species in the samples in the air. TGA was performed at a heating rate of 10 °C min^−1^ and in the temperature range of 25–950 °C.

### Electrochemical Measurements

The electrodes for NG, TiO_2‐x_@NG, and TiO_2‐x‐y_N_y_@NG were prepared by blending the active component with a conductive additive—Super P and a polyvinylidene fluoride (PVDF) binder at a ratio of 80:10:10 using N‐methylpyrrolidone as the solvent. Subsequently, the prepared mixture was applied to a copper foil via doctor blading. The coated copper foil was then placed in an oven at a temperature of 60 °C and dried thoroughly. After drying, the electrodes were fashioned into discs measuring 14 mm in diameter. The discs were subjected to overnight baking in a vacuum oven at 80 °C to eliminate any remaining moisture. Next, coin cells were assembled in an argon‐filled glove box, maintaining the water and oxygen levels below 0.01 ppm. A Li foil with a diameter of 16 mm was used as the counter electrode. A Celgard 2500 PP film was used as the separator. The electrolyte used was a 1.0 M solution of LiPF_6_ dissolved in a solution containing ethylene carbonate and diethylene carbonate in a 1:1 volume ratio. Next, 5 wt.% FEC from DodoChem was added to the electrolyte. Full cells were constructed using LiFePO_4_ (LFP) cathodes and anodes having a negative/positive (N/P) electrode capacity ratio of 1.1–1.2. In the full cells, 80 µL of the prepared electrolyte was used. The electrolyte used in the full cell was the same as that used in the half‐cell. The cathode was fabricated by mixing LFP, a conductive additive—Super P, and a polyvinylidene fluoride binder mixed at a mass ratio of 90:5:5. The prepared mixture was then evenly coated on aluminum foil and dried in a vacuum oven using the same drying procedure (at 100 °C for 10 h) as that used for fabricating the anode. The full cells exhibited a voltage between 2.4 and 3.8 V, with a 1 C rate corresponding to 170 mA g^−1^. The NEWARE BTS80 programmable battery test system was utilized for long‐term constant‐current cycling performance, rate performance, and GITT tests. The voltage window of the electrochemical performance tests ranged from 0.005 to 2 V versus Li/Li^+^. Electrochemical impedance spectroscopy and CV were performed on a CHI660E electrochemical workstation (CH Instruments, Shanghai).

## Conflict of Interest

The authors declare no conflict of interest.

## Supporting information



Supporting Information

## Data Availability

The data that support the findings of this study are available from the corresponding author upon reasonable request.
