# Supplementary material for: LiF‐Dominated SEI Formation via a Lychee‐Like Primary Interphase for Fast‐Charging Natural Graphite Anodes
Source: Small. 2025 Jul 7;21(35):2504255. doi: 10.1002/smll.202504255 (PMC12410900; doi:10.1002/smll.202504255)
Supplement: Supplementary file 1 — Supporting Information [file SMLL-21-2504255-s001.docx]

LiF-dominated SEI Formation via a Lychee-Like Primary Interphase for Fast-Charging Natural Graphite Anodes

Xiangqi Liu, Qitao Shi*, Jiaqi Wang, Junjin Zhang, Cheng Zhang, Zhipeng Wang, Luwen Li, Alicja Bachmatiuk, Yanbin Shen*, Ruizhi Yang * and Mark H. Rümmeli*

Xiangqi Liu, Jiaqi Wang, Junjin Zhang, Cheng Zhang, Zhipeng Wang, Luwen Li, Ruizhi Yang, Mark H. Rümmeli

Soochow Institute for Energy and Materials Innovation

College of Energy

Key Laboratory of Advanced Carbon Materials and Wearable Energy Technologies of Jiangsu Province

Key Laboratory of Core Technology of High Specific Energy Battery and Key Materials for Petroleum and Chemical Industry

Soochow University

Suzhou 215006, China
E-mail: yangrz@suda.edu.cn; mhr1@vsb.cz

Qitao Shi, Yanbin Shen

i-Lab, CAS Center for Excellence in Nanoscience

Suzhou Institute of Nano-Tech and Nano-Bionics (SINANO)

Chinese Academy of Sciences (CAS)

Suzhou 215123, China

E-mail: [qtshi2023@sinano.ac.cn](mailto:qtshi2023@sinano.ac.cn); ybshen2017@sinano.ac.cn

Alicja Bachmatiuk

Faculty of Chemistry

Wroclaw University of Science and Technology

Wybrzeze Wyspiarskiego 27, 50-370 Wroclaw, Poland

Alicja Bachmatiuk, M. H. Rümmeli

Electron Beam Emergent Additive Manufacturing (EBEAM Centre)

Institute of Environmental Technology (IET)

Centre for Energy and Environmental Technologies (CEET)

VSB—Technical University of Ostrava

17 Listopadu 15, 708 33 Ostrava, Czech Republic

Mark H. Rimmeli

Institute for Materials Chemistry

IFW Dresden

20 Helmholtz Strasse

Dresden 01069

Germany


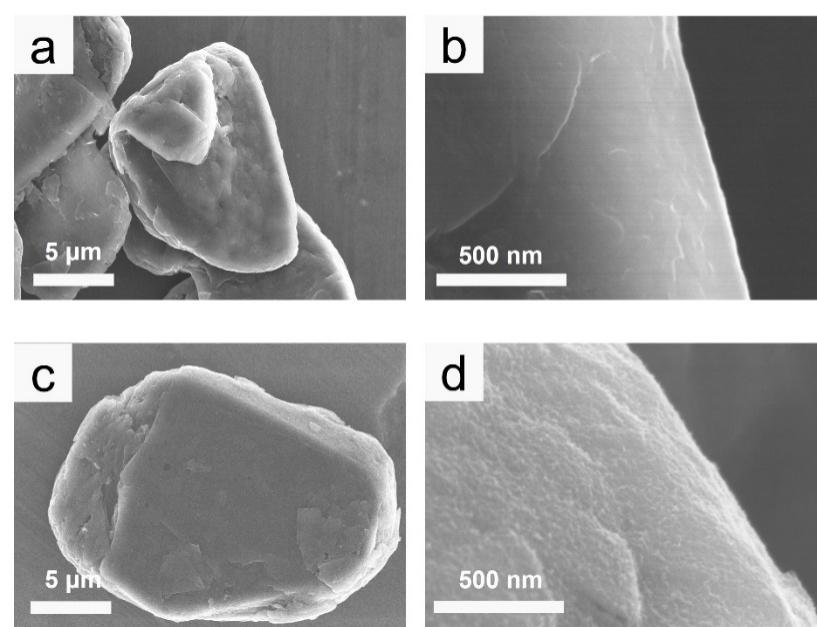


**Figure S1** SEM images of (a, b) NG and (c, d) TiO_2-x_@NG.


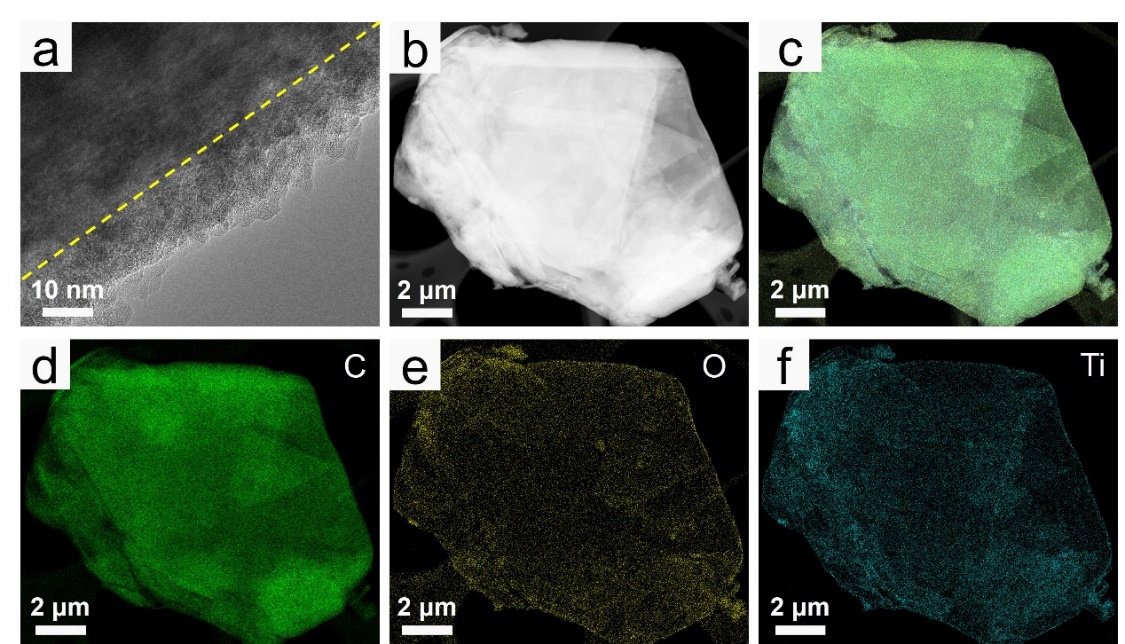


**Figure S2** (a) TEM images of TiO_2-x_@NG. (b)The STEM image taken under the high-angle annular dark-field mode and (c, d, e, f) the corresponding EDS mapping of TiO_2-x_@NG.


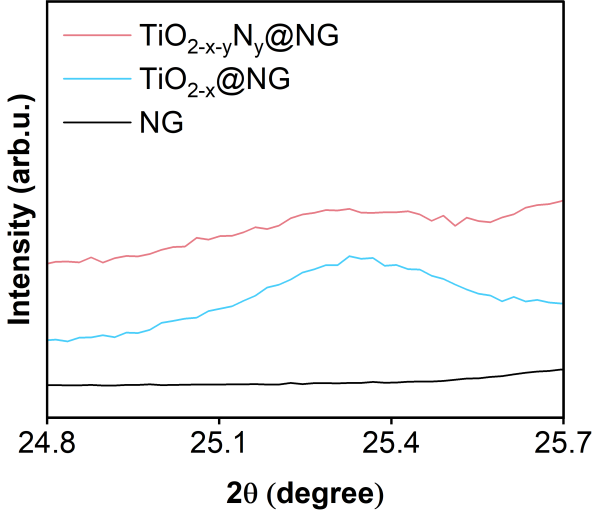


**Figure S3** XRD patterns of NG, TiO_2-x_@NG and TiO_2-x-y_N_y_@NG.


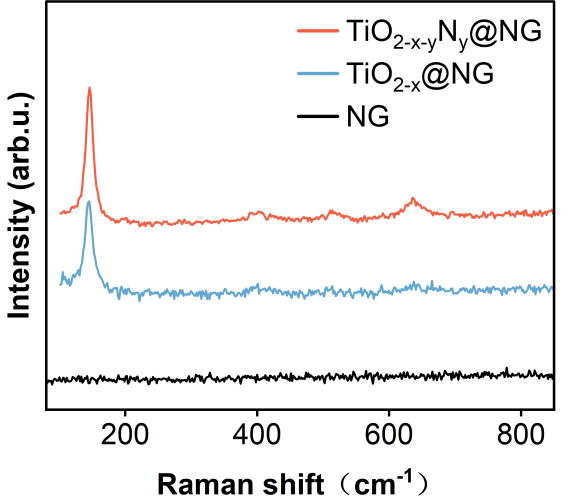


**Figure S4** Raman patterns of NG, TiO_2_@NG and TiO_2-x_N_x_@NG.


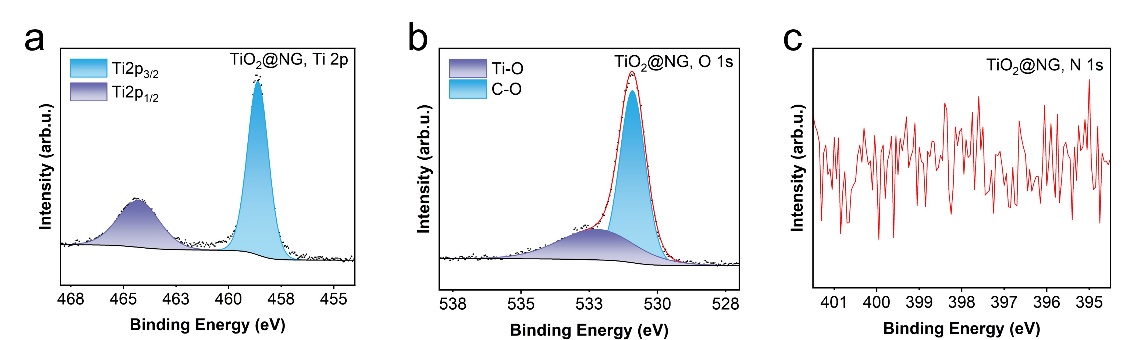


**Figure S5** XPS spectra of (a) Ti 2p, (b) O 1s, and (c) N 1s of TiO_2_@NG.


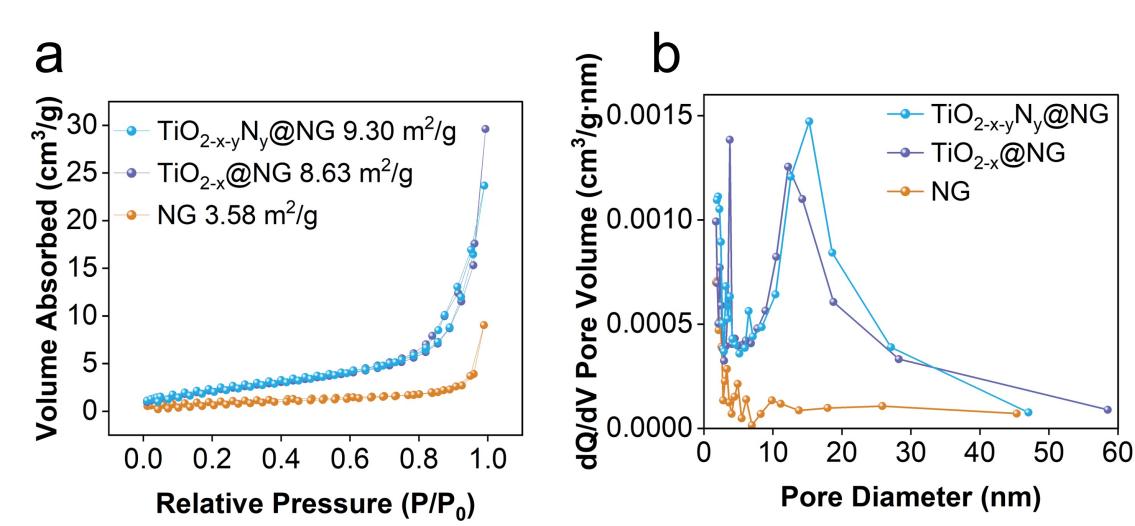


**Figure S6** (a) BET patterns and (b) pore size distributions of NG, TiO_2-x_@NG and TiO_2-x-y_N_y_@NG.


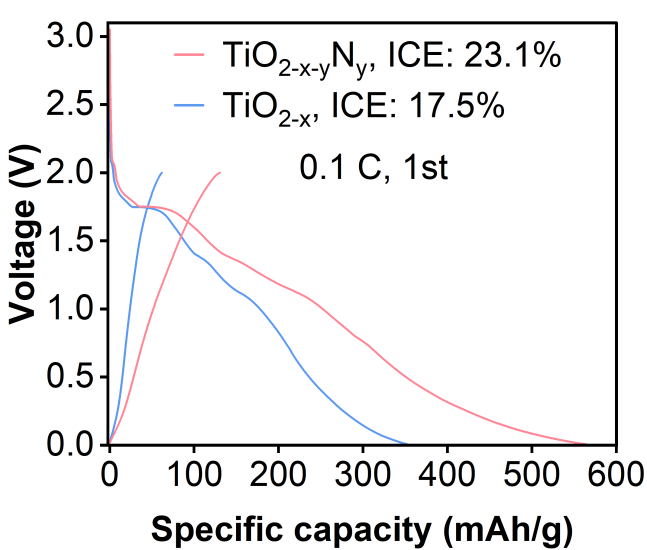


**Figure S7** Discharge-charge curves of TiO_2-x_ and TiO_2-x-y_N_y_@NG anodes at 0.1C.


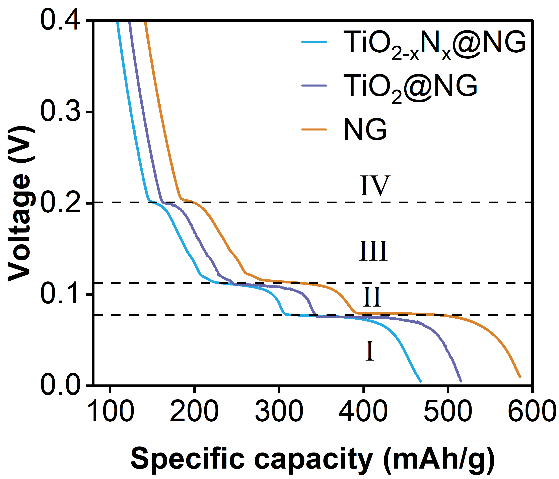


**Figure S8** Discharge-charge curves of NG, TiO_2_@NG and TiO_2-x_N_x_@NG anodes at 0.1C.


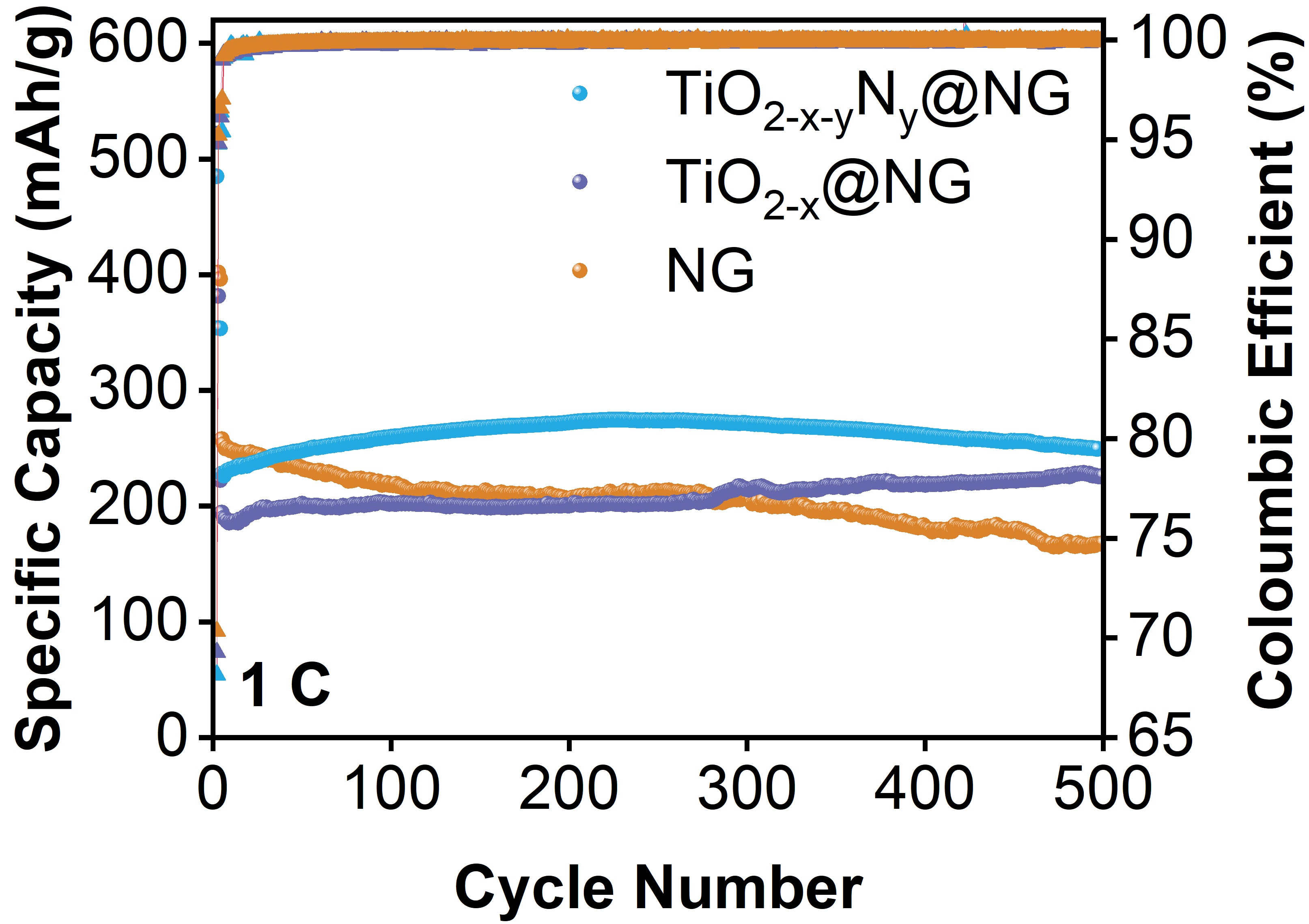


**Figure S9** The cycling performance of NG, TiO_2_@NG and TiO_2-x_N_x_@NG anodes at 1 C.


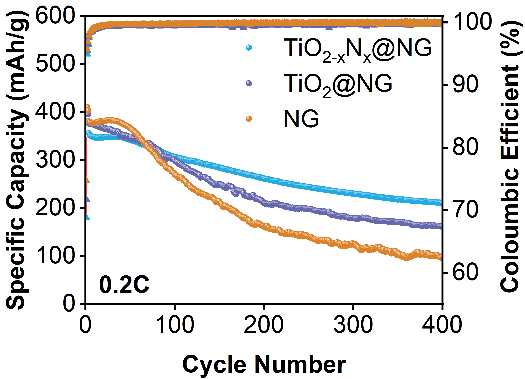


**Figure S10** The cycling performance of NG, TiO_2_@NG and TiO_2-x_N_x_@NG anodes at 0.2 C.


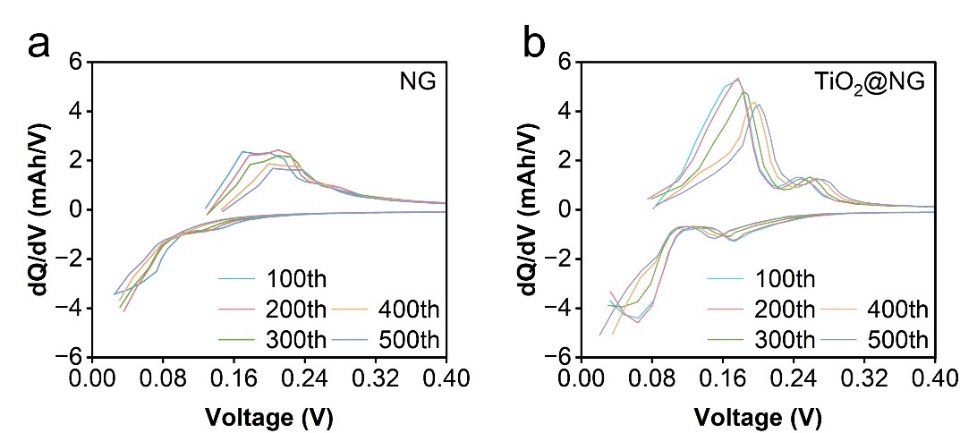


**Figure S11** dQ/dV curve of (a)NG and (b) TiO_2_@NG anodes.


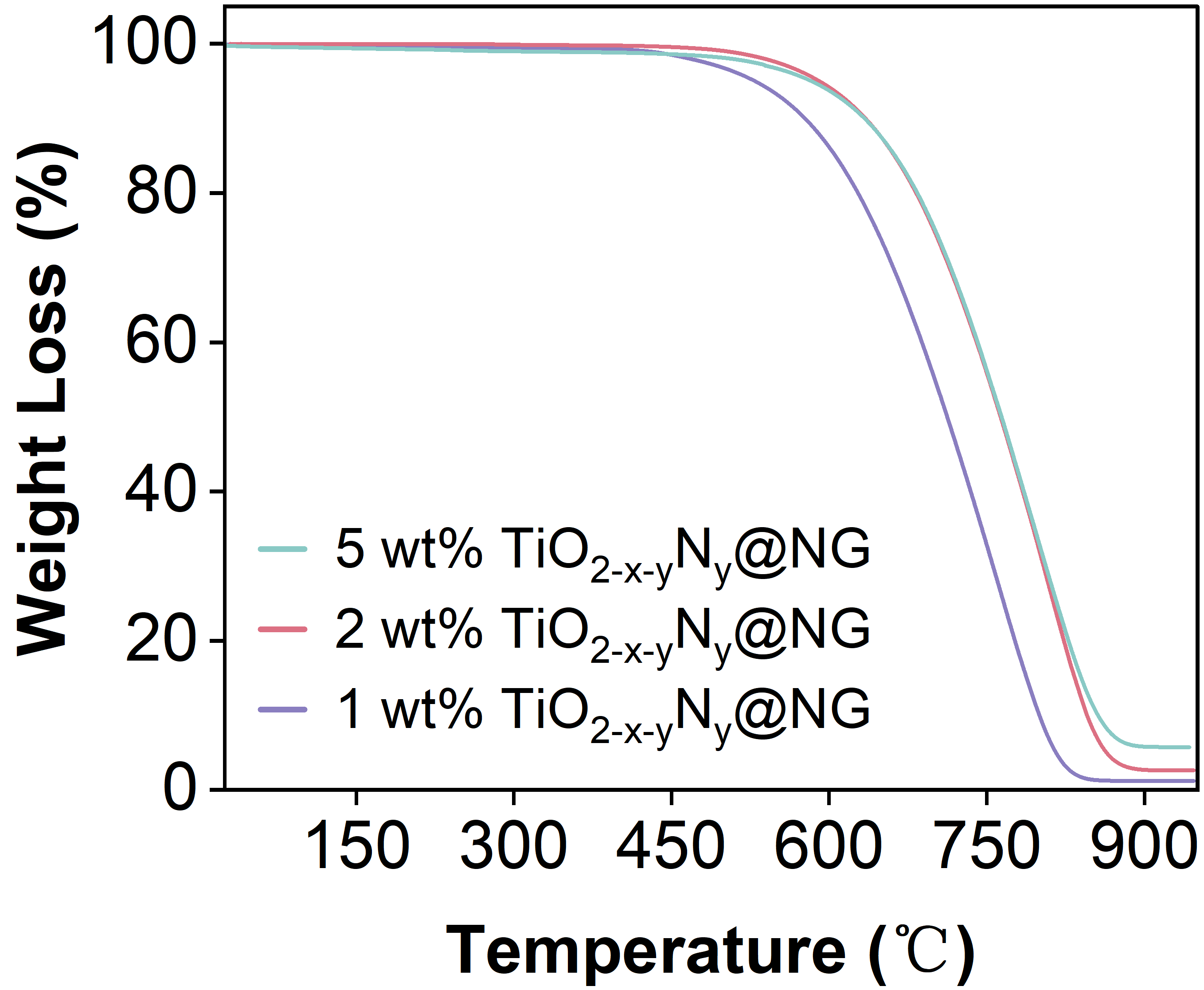


**Figure S12** TGA patterns of 5 wt% 2 wt% and 1 wt% TiO_2-x-y_N_y_@NG.


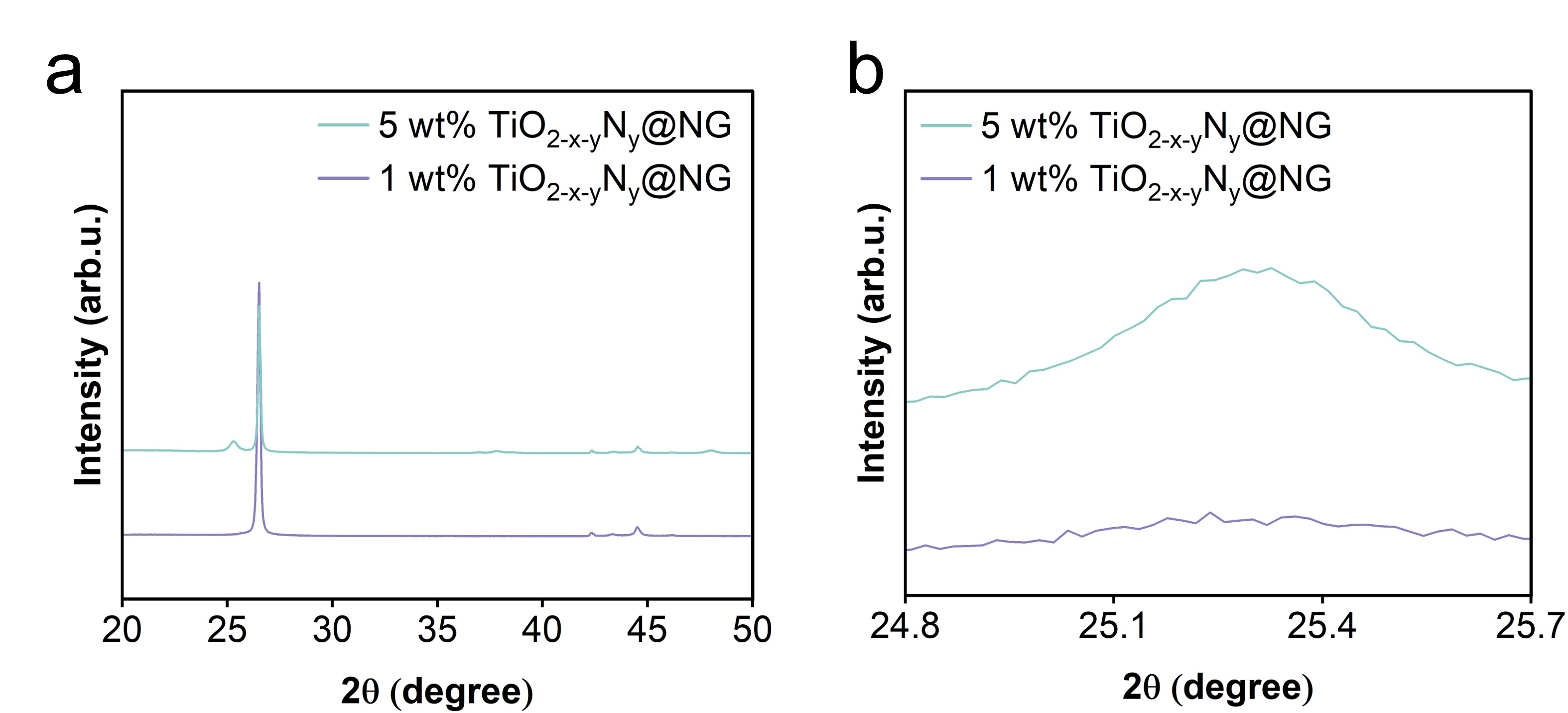


**Figure S13** XRD patterns of 5 wt% and 1 wt% TiO_2-x-y_N_y_@NG.


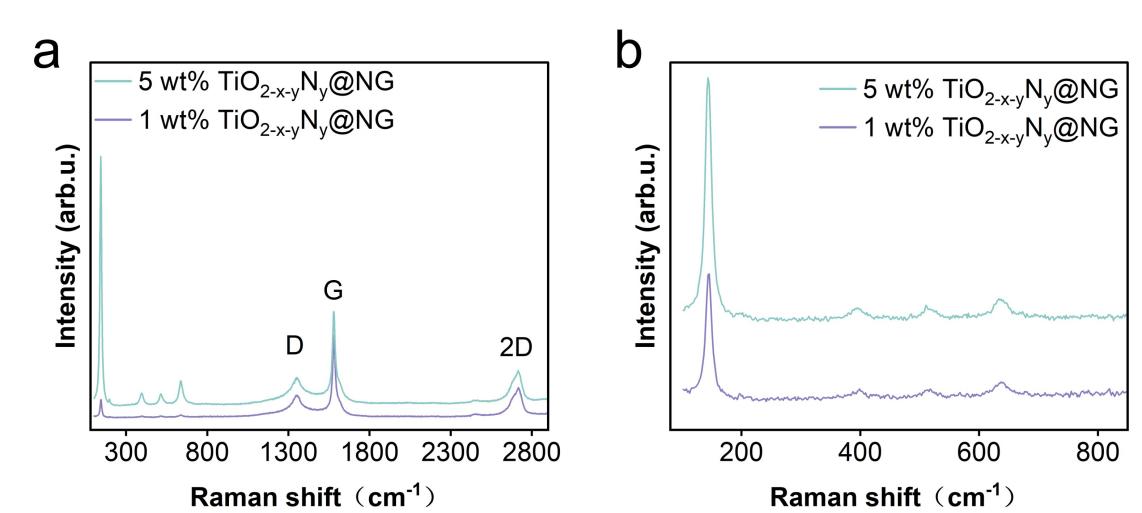


**Figure S14** Raman patterns of 5 wt% and 1 wt% TiO_2-x-y_N_y_@NG.


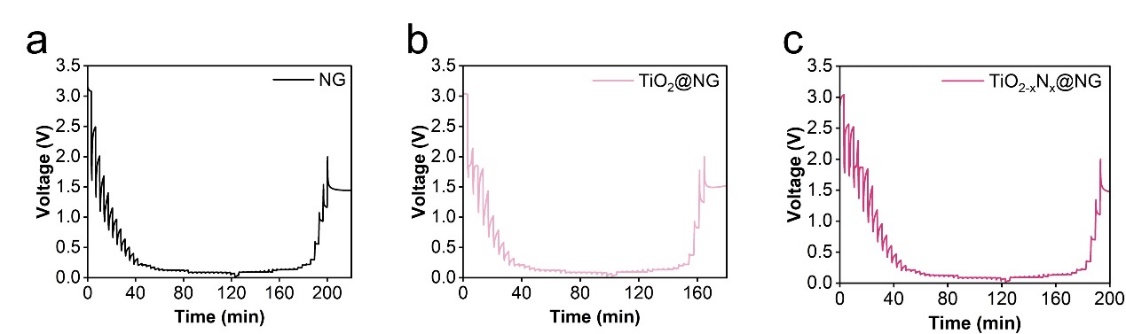


**Figure S15** GITT profiles of (a)NG, (b) TiO_2-x_@NG, (c) TiO_2-x-y_N_y_@NG anodes.


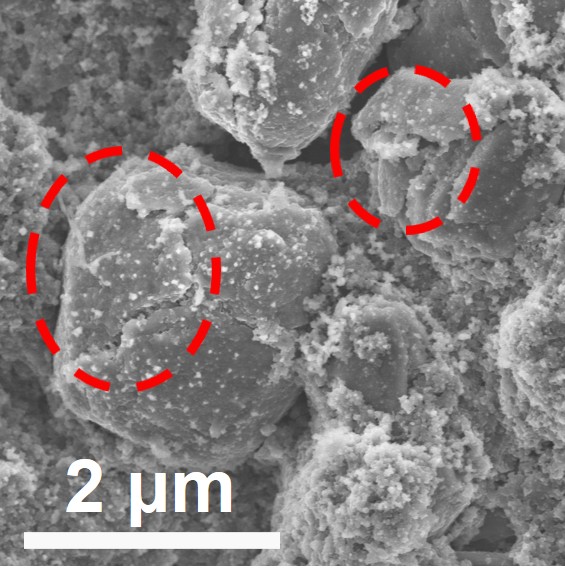


**Figure S16** SEM images of TiO_2-x_@NG anodes after 500 cycles at 1 C.

**
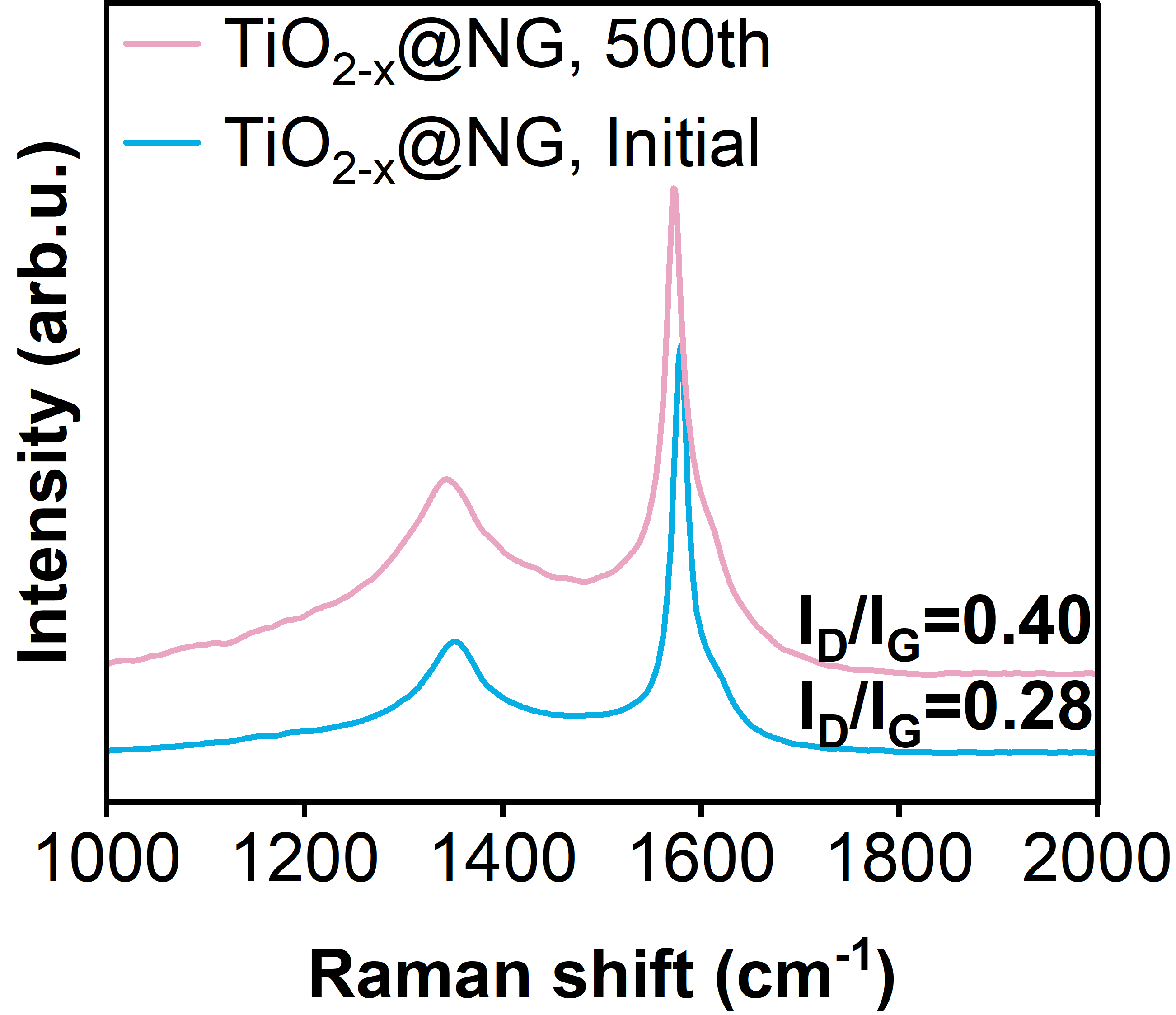
**

**Figure S17** Ex-situ Raman spectra of TiO_2-x_@NG anodes in the initial state and after 500 cycles at 1 C.


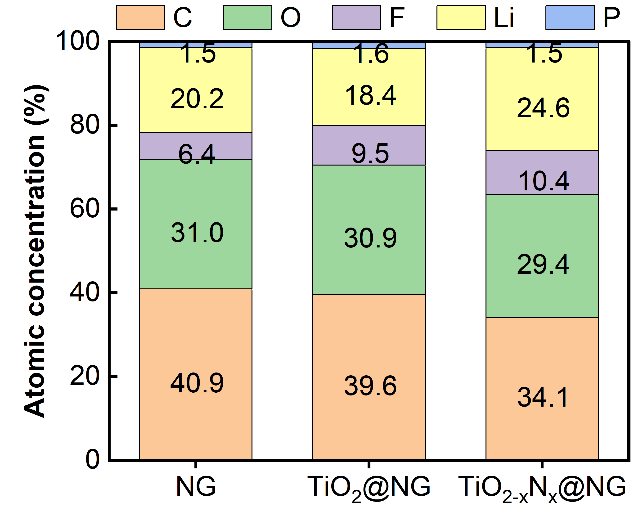


**Figure S18** Quantified atomic composition ratios of SEI layers on NG, TiO_2-x_@NG and TiO_2-x-y_N_y_@NG anodes after 500 cycles at 1C.

**
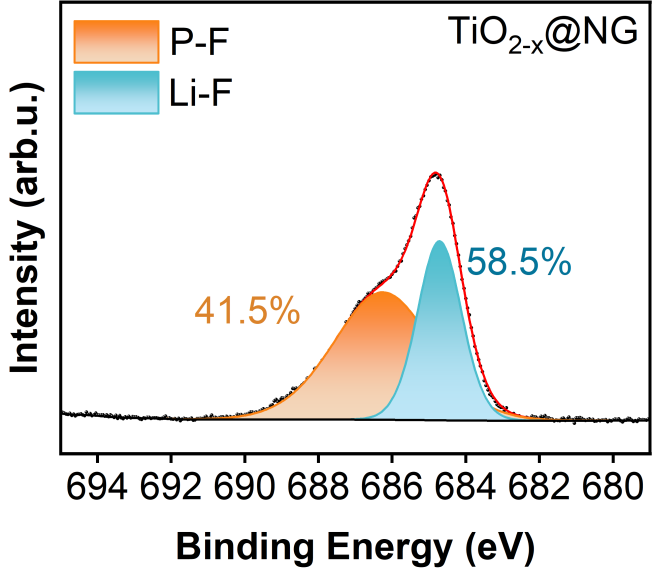
**

**Figure S19** XPS F 1s spectra of SEI layers on the surface of TiO_2-x_@NG anodes after 500 cycles at 1 C.
